# Role of emotional processing in depressive responses to sex-hormone manipulation: a pharmacological fMRI study

**DOI:** 10.1038/tp.2015.184

**Published:** 2015-12-01

**Authors:** S Henningsson, K H Madsen, A Pinborg, M Heede, G M Knudsen, H R Siebner, V G Frokjaer

**Affiliations:** 1 Center for Integrated Molecular Brain Imaging and Neurobiology Research Unit, Department of Neurology, Copenhagen University Hospital, Rigshospitalet, Copenhagen, Denmark; 2 Danish Research Centre for Magnetic Resonance, Centre for Functional and Diagnostic Imaging and Research, Hvidovre Hospital, Hvidovre, Denmark; 3 DTU Compute, Technical University of Denmark, Lyngby, Denmark; 4 The Fertility Clinic, Department of Obstetrics and Gynecology, Copenhagen University Hospital, Rigshospitalet, Copenhagen, Denmark; 5 Faculty of Health and Medical Sciences, Copenhagen University Hospital, Copenhagen, Denmark; 6 Department of Neurology, Bispebjerg Hospital, Copenhagen, Denmark

## Abstract

Sex-hormone fluctuations may increase risk for developing depressive symptoms and alter emotional processing as supported by observations in menopausal and pre- to postpartum transition. In this double-blinded, placebo-controlled study, we used blood−oxygen level dependent functional magnetic resonance imaging (fMRI) to investigate if sex-steroid hormone manipulation with a gonadotropin-releasing hormone agonist (GnRHa) influences emotional processing. Fifty-six healthy women were investigated twice: at baseline (follicular phase of menstrual cycle) and 16±3 days post intervention. At both sessions, fMRI-scans during exposure to faces expressing fear, anger, happiness or no emotion, depressive symptom scores and estradiol levels were acquired. The fMRI analyses focused on regions of interest for emotional processing. As expected, GnRHa initially increased and subsequently reduced estradiol to menopausal levels, which was accompanied by an increase in subclinical depressive symptoms relative to placebo. Women who displayed larger GnRHa-induced increase in depressive symptoms had a larger increase in both negative and positive emotion-elicited activity in the anterior insula. When considering the post-GnRHa scan only, depressive responses were associated with emotion-elicited activity in the anterior insula and amygdala. The effect on regional activity in anterior insula was not associated with the estradiol net decline, only by the GnRHa-induced changes in mood. Our data implicate enhanced insula recruitment during emotional processing in the emergence of depressive symptoms following sex-hormone fluctuations. This may correspond to the emotional hypersensitivity frequently experienced by women postpartum.

## Introduction

Mood disorders are among the most common causes for disability and constitute a major and severe public health problem.^1^ The prevalence is twice as high in women relative to men.^2^ Postpartum depression, premenstrual dysphoric disorder (PMDD) and major depression with onset in the menopausal transition phase are examples of mood disorders coinciding with fluctuations, or a rapid decline, in sex-steroid hormone levels. The mechanisms underlying these phenomena are not well understood, but may be related to neurobiological effects of changes in ovarian sex steroids, in particular estradiol.^3, 4^

Altered processing of emotional stimuli has been recognised as a core cognitive abnormality in mood disorders, including major depression, postpartum depression and PMDD.^5, 6, 7, 8^ Comparisons across menstrual cycle phases, associated with subtle variations in sex-steroid hormone levels, that is, the follicular versus luteal phases, have also suggested phase-specific variations in emotional processing.^8, 9, 10, 11, 12^ However, those studies offer no consensus with respect to the pattern of brain regions recruited differently across menstrual cycle phases. Further, chronic states of ovarian sex-steroid hormone downregulation, that is, with hormonal contraception, have been associated with blunted amygdala responses to negatively valenced emotional stimuli suggesting that such longer-term ovarian hormone deprivation might evoke changes in emotional processing.^13^

In addition to the coupling between sex-steroid hormones, emotional processing and mood, this study is motivated by frequent reports of a relationship between serotonergic transmission and emotional processing^14, 15, 16^ since sex steroids and serotonin interact. This interaction is supported in particular by rodent and non-human primate work of Bethea *et al.*^17^

Here we studied emotional processing before and after manipulating ovarian sex-steroid hormone production by intervention with the gonadotropin-releasing hormone agonist (GnRHa) Goserelin. When administered continuously, GnRHa initially stimulates and subsequently, after ~2 weeks, downregulates ovarian hormone production, in particular estradiol, by desensitising GnRH receptors at the level of the pituitary gland. As this intervention induces a transient fluctuation in ovarian hormones in healthy women, it can serve as a model for studying the neurobiological correlates of such a fluctuation and its potential coupling to behavioural or psychological responses. Using blood−oxygen level dependent (BOLD) functional magnetic resonance imaging (fMRI), we mapped functional brain activation in emotion-related regions during implicit (gender labelling) emotional processing of faces expressing fear, anger, happiness and no emotion. Functional brain activity was measured with fMRI at baseline and after intervention with either GnRHa or placebo. The post-intervention session was placed shortly after the biphasic GnRHa response in the early suppression phase as motivated by studies documenting that risk for depression in perimenopause is particularly associated with fluctuations in estradiol^4^ and peaks early (day 10–19) postpartum.^18^ We hypothesised that GnRHa would induce changes in depressive symptoms in a manner dependent on estradiol changes and emotion-elicited brain activity.

## Materials and methods

### Participants, intervention and assessment timing

Participants were recruited by internet advertisements. Inclusion criteria comprised regular menstrual cycles (duration 23–35 days) for at least 3 months before inclusion, normal hormone blood tests (including follicle-stimulating hormone, thyroid-stimulating hormone and androgens) and no history of neurological, gynaecological or psychiatric disorders, including PMDD, as assessed by Schedules for Clinical Assessment in Neuropsychiatry (SCAN 2.1) interview, as well as neurological and gynaecological examinations including a transvaginal ultrasound of the uterus and ovaries. Participants were enroled in the baseline assessment program at a follicular phase cycle day and booked for their second ultrasound examination at cycle day 21 or 22 to assure ovulation and secure intervention timing to the midluteal phase as further detailed in Frokjaer *et al.*^19^

Sixty-three healthy women participated in this randomised, placebo-controlled and double-blinded intervention study. Participants received 3.6 mg GnRHa (Goserelin) implant (*n*=31) or saline injection (*n*=30) in a natural cycle during the midluteal phase (cycle day 22.7±2.7). Two participants did not complete follow-up because of anovulation and pregnancy, respectively. Three did not complete the follow-up MRI session because of technical issues or personal matters. Two participants were excluded from the placebo group owing to faulty cycle timing of the MRI scan and failure to comply with the task, respectively. Thus valid fMRI data from both sessions were available for 56 women (age 24.3±5.0 years), 30 in the GnRHa group and 26 in the placebo group.

**Figure 1:**
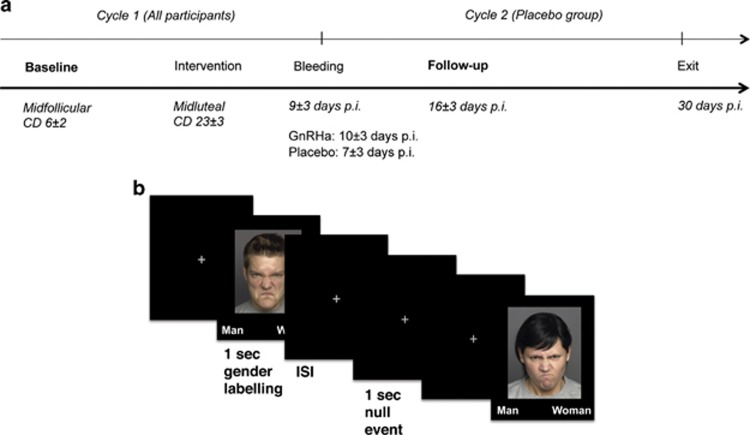
(**a**) Timeline of the study program. Valid fMRI, estradiol and Hamilton depression rating scale (17 items) data for the baseline and follow-up sessions were available for 29 subjects in the GnRHa group and 26 in the placebo group. Estradiol and Hamilton depression scores were collected 0±2 days within the fMRI day. (**b**) Example of the mode of presentation from a block of angry faces. CD, cycle day; fMRI, functional magnetic resonance imaging; GnRHa, gonadotropin-releasing hormone agonist; ISI, inter-stimulus interval; p.i., post intervention.

Baseline fMRI was acquired in the midfollicular phase (cycle day 6.0±2.3) when ovarian hormone levels are most stable and the time elapsed since the postovulatory estradiol drop is maximal. Follow-up fMRI was acquired post bleeding at a time point late enough to allow the GnRHa group to have entered their early ovarian suppression phase and the placebo group to be in the follicular phase (16.0±3 days after intervention). This design allowed for all researchers to be blinded. See Figure 1a for an outline of the time points. The participants also performed other tasks in the scanner: the first fMRI run comprised T1-weighted imaging, resting state fMRI, a word memory fMRI paradigm, T2-weighted imaging and diffusion tensor imaging. The second fMRI run, acquired after a 40 min break, comprised the emotional face fMRI paradigm reported here and a gambling fMRI paradigm. Hamilton 17-item depression rating scale and plasma concentrations of estradiol and progesterone were collected within 0±2 days from the fMRI session date at baseline and follow-up. Estradiol and progesterone levels in serum were determined as specified in ref. 19. Valid data on these measures were available for 29 and 26 subjects, respectively. The study was registered and approved by the local ethics committee (H-2-2010-108). After complete description of the study, written informed consent was obtained from all participants.

### Experimental fMRI design

At baseline and follow-up, participants performed an emotional face gender-labelling task, see ref. 20 and Figure 1b. Subjects were to indicate the gender of photographs of faces with either fearful (F), angry (A), happy (H) or neutral (N) expressions. The different conditions were presented in blocks of six events, consisting of the block-specific expressions or null events (fixation crosses, one to three per block, pseudo-randomly intermixed). Events lasted for 1 s and were interspersed with inter-stimulus fixation crosses presented for 1 s. The gender response button rule (left or right for woman or man) was counterbalanced across subjects. Participants were instructed to answer as fast and as correct as possible. The testing was split up in two 7-min-long runs, each containing seven blocks of each type. All photographs were presented only once. E-prime software (Psychological Software Tools, Pittsburgh, PA, USA) was used for stimulus presentation and response recordings.

### Behavioural data analysis

Behavioural data were analysed using statistical parametric mapping software package (SPSS, version 18, Chicago, IL, USA). Potential effects of different emotional expressions on accuracy and reaction time (RT) at baseline and at post intervention were examined by means of two separate analysis of variance with emotion (F, A, H and N) as within-subject factor (for the full sample). The intervention-related changes in accuracy and RT were assessed using a two-way repeated measures analysis of variance with emotion (F_post−pre_, A_post−pre,_ H_post−pre_, N_post−pre_) as within-subject factor and group (GnRHa versus placebo) as between-subject factor. The Greenhouse-Geisser method was used to correct for non-sphericity when appropriate.

### Image acquisition

Images were acquired on a 3T Verio scanner with a 32-channel head array coil (Siemens, Erlangen, Germany). For BOLD fMRI, a gradient echo based T2*-weighted gradient echo-planar imaging sequence was used with a repetition time of 2.15 s, echo time of 26 ms, flip angle of 78°, and 42 slices with a slice thickness of 3 mm with twofold acceleration using generalised autocalibrating partially parallel acquisitions. The echo-planar imaging sequence was optimised for signal recovery in orbitofrontal cortex.^21^ A total of 198 whole-brain volumes were acquired in each session.

### Imaging data analysis

The functional images were realigned, and normalised to an echo-planar imaging template in Montreal Neurological Institute stereotactic space using default SPM8 settings. The normalised images were smoothed using an isotropic 8 mm Gaussian kernel. Analyses were conducted in SPM8.

For each voxel in the brain, a general linear model was constructed to model event-related changes in the BOLD signal. At the within-subject level, five event types were defined for each fMRI session, which corresponded to the presentation onsets for F, A, H and N faces, as well as null events. RTs were modelled in a separate regressor in order to control for differences in RT between different emotions. Incorrect answers were excluded. The within-subject model also included 40 nuisance regressors of no interest to account for variance in regional BOLD signal changes caused by physiological noise, including head movement and measurements of heart beat and respiration.^22, 23^

Choosing a task where emotional faces could be contrasted with N faces enabled isolation of emotion-specific brain activity. Within the first-level general linear model, we specified 3T-contrasts that captured differences in the BOLD response to emotional as opposed to N images (F−N, A−N and H−N, used as acronyms for the contrasts of interest hereafter) for each participant, as well as contrasts comparing the post-intervention images with the pre-intervention images (for example, post(F−N)−pre(F−N)). Individual contrast images were entered into second-level analyses for within-group and between-group statistical comparisons. On the group-level (GnRHa versus placebo), the first contrasts were used to investigate the differential activation change from pre to post in the two groups in a flexible factorial design with group as between-subject factor and pre/post as within-subject factor. Within the GnRHa group the latter contrasts were used to investigate correlations between measures of estradiol and depressive symptoms changes from baseline on the one hand, and brain activity changes, on the other, in one-sample *t*-tests (two sided). These analyses were contingent upon active intervention, which introduced the expected biphasic estradiol change from baseline, and a main effect of group on changes in Hamilton score.^19^

In line with results from previous studies,^24, 25^ there was no significant test−retest correlation for amygdala activity in the placebo group for any of the contrasts of interest (correlation coefficients <0.3). This was the case also for insula activity. Because of this lack of significant test−retest correlation, and because of previous findings of absolute levels of estradiol being of less importance for mood than changes in levels,^3^ we also considered it appropriate to test for the main effect of group (GnRHa versus placebo) cross-sectionally at post intervention, as well as for a potential relationship between activity in the post-GnRHa session and estradiol and depressive symptoms changes, that is, not accounting for the baseline fMRI measures. In this way we could also test if the baseline fMRI measures were driving the observed effects. The relationship between post-GnRHa depressive symptoms and post-GnRHa brain activity was also investigated. When appropriate, the beta values of significant peaks for each subject were extracted in order to plot the value against changes in estradiol or depression scores.

The general significance level was set at *P*<0.05 for activation clusters after correction for multiple non-independent comparisons using family wise error correction. Our *a priori* primary region of interest (ROI) was the amygdala, due to the well-documented involvement in mood.^5, 6, 7^ Secondary ROIs were the medial prefrontal cortex, including the medial orbitofrontal cortex and the anterior cingulate cortex, the ventrolateral prefrontal cortex (the sum of the Frontal_Inf_Tri and Frontal_Inf_Orb) as well as insula, all defined by the Pick atlas,^26^ based on previous reports underscoring their relevance in emotional processing.^27, 28, 29, 30^ We applied small volume correction (SVC) using SPM8 for each of the ROIs to obtain family wise error-corrected *P*-values based on Gaussian random fields. Trends are reported at the cluster-forming threshold, which was set to *P*_unc_<0.001 (unc, uncorrected) for clusters consisting of at least five voxels.

### Analyses of estradiol measures

Estradiol decline ‘dose-response' analyses were considered appropriate in the GnRHa group only. Changes in estradiol levels were treated as Log_2_Estradiol_post_–Log_2_Estradiol_pre_ because of the lack of normal distribution for the raw delta values, as previously validated.^19^ Values below detection limit (*n*=4) were imputed to the lower detection limit: 0.04 nmol l^–1^.

## Results

### Behavioural results

As previously reported for the full sample,^19^ Goserelin initially increased and subsequently reduced estradiol levels (from 0.18±0.09 to 0.06±0.04 nmol l^–1^), and led to an increase in depressive symptoms on the Hamilton 17-item scale relative to placebo (from 1.2±1.6 to 3.0±2.6, *P*<0.001). There was no net decline in progesterone levels from baseline to follow-up (see ref. 19). The depressive symptoms were subtle except in two participants who met the clinical criteria of a mild depressive state. The magnitude of net estradiol decrease in response to GnRHa and the emergence in depressive symptoms were positively correlated (Pearson correlation= 0.39, *P*=0.04).

**Figure 2:**
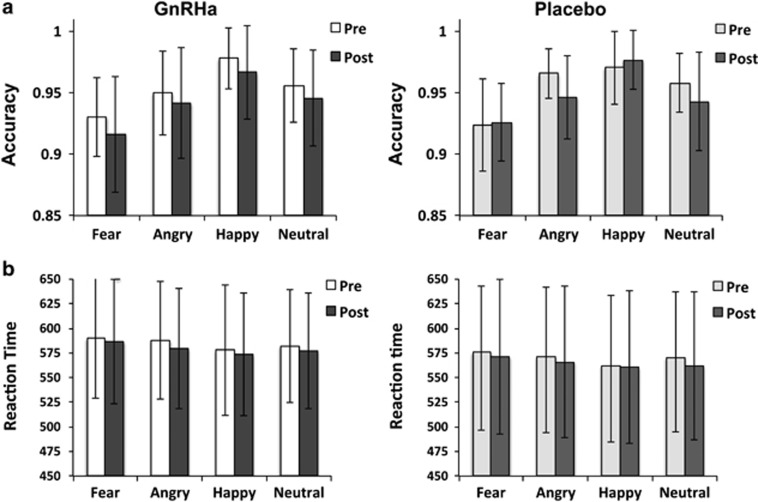
(**a**) Accuracy. (**b**) Reaction times for the GnRHa (*n*=30) and placebo (*n*=26) groups pre- and post-intervention. Both pre- and post-intervention, facial emotion was a significant within-subject factor on measures of accuracy and RT (*P*-values<0.001, F_Accuracy_Pre_=41.8 d*f*=(2.8, 153.5), F_RT_Pre_=8.6, d*f*=(2.8, 152.8), F_Accuracy_Post_=44.9, d*f*=(2.8, 153.8), F_RT_Post_=6.6, d*f*=(2.6, 147.3)). Accuracy was lower for F and higher for H faces compared with N, whereas, as expected, RTs were slower for F and A faces compared with N and H faces (**a** and **b**). The analyses of intervention-related changes in accuracy revealed a group by pre−post interaction, explained by an unexpected accuracy reduction (from baseline) for A and N faces in the placebo group. *Post hoc* analyses showed that for angry faces there was also a significant baseline difference (placebo>GnRHa) between groups in accuracy (*P*_unc_=0.04) that may partly have driven the interaction effect (**a**). There was no Group by pre/post interaction on RT, indicating RTs were unaffected by GnRHa (F=0.39, d*f*=(2.8, 150.7), *P*=0.74). Notably, there was a slight non-significant decrease in RT for both groups on the post-intervention session. A, angry; F, fearful; GnRHa, gonadotropin-releasing hormone agonist; H, happy; N, neutral.

Both pre- and post-intervention, facial emotion was a significant within-subject factor on measures of accuracy and RT (Figures 2a and b). The analyses of intervention-related changes in accuracy revealed a group by pre−post interaction (F=3.0, d*f*=(3.0, 160.2), *P*=0.03), explained by an unexpected accuracy reduction (from baseline) for A and N faces in the placebo group (Figures 2a and b). Incorrect answers were excluded and RT was modelled in a separate regressor for the fMRI analyses.

### Imaging results

#### Main effects of treatment group

There was no significant main effect of treatment group (GnRHa versus placebo) nor a group by pre/post-intervention interaction for the neural response to F, A or H faces, as compared with N in any ROIs. No trends were observed for the primary ROI amygdala, but for secondary ROIs a trend (*P*_unc_<0.001) for a larger increase in left perigenual anterior cingulate cortex activity in the GnRHa group, compared with the placebo group, was present for F and A faces (*xyz*=(−14 38 −4), peak *Z*-scores=3.4). When considering only the post-intervention session, relative to the placebo group, the GnRHa group tended (*P*_unc_<0.001) to show larger activity in the left anterior insula to A faces post intervention (*xyz*=(–34 26 8), peak *Z*-score=3.3, *P*_SVC_=not significant).

#### Correlations with depressive responses to GnRHa intervention

Within the GnRHa group, individual increases in depressive symptoms from baseline correlated significantly with an increased neural response in the insula ROI to F (left) and H (right) facial expressions (F−N: *xyz*=(–32 16 0), peak *Z*-score=3.6 *P*_SVC_<0.05, H−N: *xyz*=(34 32 6), peak *Z*-score=4.0 *P*_SVC_<0.001), with similar but weaker trends for H faces in the contralateral hemispheres (H−N *xyz*=(–26 22 –2), peak *Z*-score =3.7, *P*_unc_<0.001) and also a trend (*P*_unc_<0.001) bilaterally for A faces (A−N: *xyz*=(–26 26 –2), peak *Z*-score=3.6 and *xyz*=(34 32 4), peak *Z*-score=3.3 *P*_SVC_-values=not significant). There was also a trend for a correlation between depressive symptom increase and increased activity of the right amygdala to H faces (*xyz*=(28 4 –16), peak *Z*-score=3.3, *P*_SVC_=0.06). See Table 1 for details.

**Figure 3:**
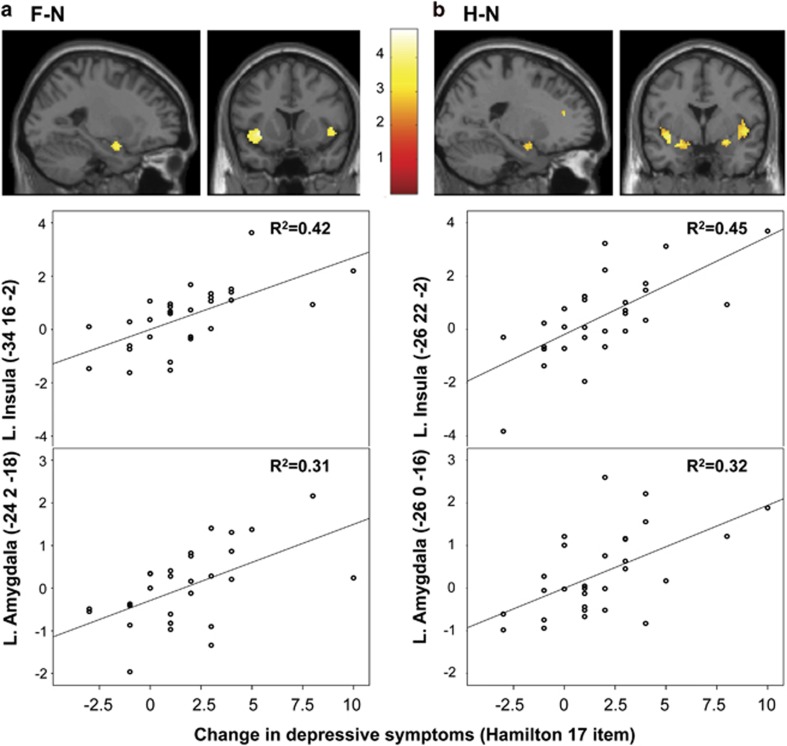
Correlations in the GnRHa group (*n*=29) between depressive symptom increase and activity in the insula and amygdala post-GnRHa to (**a**) fearful and (**b**) happy faces, respectively, as compared with neutral faces. The mask used for visualisation includes the five bilateral ROIs. GnRHa, gonadotropin-releasing hormone agonist; ROIs, region of interests.

In the placebo group and for all of the three contrasts, there was no significant correlation between extracted activity within the amygdala and insula pre- and post-intervention (Pearson correlation coefficients <0.3). In order to exclude that baseline measures drove our results, an additional analysis based on neural activity in the post-GnRHa session only was conducted. This confirmed that the increase in depressive symptoms correlated with F- and H-elicited activity in bilateral insula (F−N: *xyz*=(–34 16 –2), peak *Z*-score=4.1, *P*_SVC_<0.001 and *xyz*=(32 36 0), peak *Z-*score=4.1, *P*_SVC_<0.05, Figure 3a, H−N: *xyz*=(–26 22 –2), peak *Z*-score=4.4, *P*_SVC_<0.001 and *xyz*=(32 22 –2), peak *Z*-score=4.0, *P*_SVC_<0.001, Figure 3b), and further correlated with F- and H-elicited activity in the amygdala (F−N: *xyz*=(–24 2 –18), peak *Z*-score=3.5, *P*_SVC_<0.05, Figure 3a, H−N: *xyz*=(–26 0 –16), peak *Z*-score=3.7, *P*_SVC_<0.05, Figure 3b and *xyz*=(28 2 –14), peak *Z*-score=3.5, *P*_SVC_<0.05). There was a similar trend (*P*_unc_<0.001 for A faces within the right and left insula (*xyz*=(32 24 0), peak *Z*-score=3.6 and *xyz*=(–26 26 –4), peak *Z*-score=3.3) but not in the amygdala (*P*_unc_>0.05). See Table 1 for details. The correlations between insula and amygdala activity and changes in depressive scores remained significant also after adjusting for changes in estradiol levels.

When considering the relationship between brain activity at the post-GnRHa session and absolute Hamilton scores (as opposed to changes from baseline) for depressive symptoms at the post-GnRHa session, similar patterns emerged, with significant correlations in bilateral insula and left amygdala (Table 1). In addition, happiness-elicited activity in the supragenual anterior cingulate cortex correlated with depressive symptoms (Table 1). Changes in depressive symptoms correlated with depressive symptom scores at the post-GnRHa session (Pearson correlation=0.84, *P*<0.001).

#### Correlations with estradiol decline

Within the GnRHa group, the net decline in estradiol levels across the intervention period tended to correlate (*P*_unc_<0.001) with an altered response of the left ventrolateral prefrontal cortex to F faces (*xyz*=(–42 18 16), peak *Z*-score=3.7, *P*_SVC_=0.12) from baseline. When considering the post-GnRHa session only, no significant correlations with the magnitude of the estradiol decline (*P*_unc_>0.001) were revealed. This was the case also in a complete case analysis where the four cases with estradiol values below detection limit were omitted, as well as when estradiol measures were treated on the absolute scale.

#### Imaging results at baseline

Pre-intervention, for the full sample, A, but not F, faces elicited significantly stronger left amygdala activity than N faces (*xyz*=(–28 2 –22), peak *Z*-score=3.6, *P*_SVC_<0.05). Pre-intervention, H faces elicited significant right anterior insula activity (*xyz*=(44 18 –4), peak *Z*-score=3.5, *P*_SVC_<0.05), with a trend (*P*_unc_<0.001) in the left insula as well as for F and A faces. None of the ROIs displayed a significant correlation between activity to any of the emotional faces and baseline estradiol levels (across groups). However, there was a trend (*P*_unc_<0.001) for a positive correlation between baseline estradiol levels and activity to F and A faces in bilateral ventrolateral prefrontal cortex (F−N: *xyz*=(–46 48 2), peak *Z*-score=3.7, *P*_SVC_=0.07; A−N: *xyz*=(–52 36 22), peak *Z*-score=3.6, *P*_SVC_=0.09 and *xyz*=(56 40 12), peak *Z*-score=3.8, *P*_SVC_=0.06), with a similar trend for H faces.

## Discussion

The ovarian sex-steroid hormone manipulation affected processing of emotional faces in a manner dependent on depressive responses to the intervention. Women with a larger GnRHa-induced depressive response had a larger increase in emotion-elicited insula activity irrespective of emotional valence. When only considering the post-GnRHa fMRI session, apart from confirming the insula finding, also amygdala recruitment was associated with a larger depressive response. These findings were not driven by baseline conditions, and the magnitude of the GnRHa-induced net estradiol decline did not account for the effects.

Our findings of changes in emotional processing may either be directly pharmacologically driven or secondary to the (sub)clinical depressive symptoms that may emerge in particularly sensitive individuals. The absence of an association between GnRHa-induced estradiol decline and emotion-probed brain activity that map onto changes in depressive symptoms supports the latter. However, it does not necessarily exclude that hormone fluctuations mediate the current findings. Since GnRHa induces a biphasic ovarian hormone response, where the estradiol decline is preceded by an augmentation, variability in the magnitude of the initial augmentation may be of importance, as may inter-individual variability in sensitivity either to the augmentation or to the subsequent estradiol drop.^12^ If such increased estradiol sensitivity would translate to an increased bottom-up engagement of the insula and amygdala to emotional stimuli, our findings would be compatible with the notion that the depressive response is a consequence of the change in emotion processing. Notably, previous studies in women show differences in sensitivity to fluctuations in estradiol to be critical for the development of a depressed state; subjects with a history of depression have increased risk of depression as a function of the estradiol fluctuations during menopausal transition,^31^ and, estradiol sensitivity in late pregnancy, where sex-steroid levels are massively increased, predicts postpartum depression.^12, 32, 33^ Thus, inter-individual differences in the sensitivity to the same estradiol changes may be of importance for the interpretation of the current findings. Future studies should elucidate the importance of an estradiol flare and study relevant high-risk populations to further explore the importance of estradiol sensitivity, for example, by including biomarkers of sensitivity to sex steroids.

Our data suggest that, with increased depressive symptoms, insula engagement when processing negative stimuli changed in the same direction as that of positive stimuli. The fact that the engagement of insula was independent of emotional valence is well in line with previous studies suggesting that insula reacts to both positive and negative stimuli.^34, 35^ A core function of insula, in particular the anterior portion, seems to be to facilitate bottom-up detection of salient events that informs additional processing and the generation of appropriate behavioural responses.^36^ The positive correlation between insula activity change to emotional stimuli and increase in depressive symptoms we observed is consistent with some^34, 37, 38^ but not all^39, 40, 41^ studies. Previous studies suggesting that insula function is sensitive to sex-steroid hormone changes offer no consensus on directions. In healthy individuals, left insula activity has been noted to be decreased in response to negative emotional stimuli in the luteal phase of the menstrual cycle.^42^ In contrast, a study in PMDD patients points towards enhanced insula activation during response inhibition in the symptomatic phase (luteal phase),^43^ which is well in line with our observations.

We speculate that an increased insula engagement in processing of salient stimuli might represent a mechanism by which sex-steroid hormone fluctuations can trigger disturbed affective processing and hence influence mood in vulnerable individuals. It is possible that hyperreactivity to emotional salience is a correlate to the postpartum blues state that occurs in most women 3–5 days postpartum, which is characterized by dysphoric mood, crying, mood lability, anxiety, irritability and a feeling of easily being overwhelmed with emotions.^44^

When only the post-GnRHa session was considered, the observation of higher activity in subjects with a larger increase in depressive symptoms extended beyond the insula, and also included our primary ROI, the amygdala. Thus, apparently, women who mood-wise were more vulnerable to GnRHa sex-hormone manipulation displayed increased amygdala and insula involvement in processing salient stimuli, independent of stimulus valence. Several studies support that amygdala react to both negative and positive stimuli.^45, 46, 47, 48, 49^ Hyperactivity of the amygdala has also been reported in the luteal, as compared with the follicular, phase of the menstrual cycle in healthy women when estradiol levels are decreasing.^6, 11, 50^ In contrast to our GnRHa-related findings, blunting of the amygdala response to aversive stimuli has been reported to correlate with depression severity in manifest postpartum depression.^7^ Since our model best matches the onset of a depression and our participants only displayed subtle and primarily subclinical depressive symptoms, our findings are not directly comparable to the latter findings reported by Moses-Kolko *et al.*^7^ However, our findings are in line with the meta-analysis of Groenwold *et al.*^5^ that showed a correlation between depressive response and amygdala activity elicited by fearful stimuli, and suggest that such an effect is independent of emotional valence. With regard to the increased amygdala activity to happy faces in subjects with more GnRHa-induced depressive symptoms, no clear consensus on potential differences between controls and patients with major depression disorder exists.^40, 51, 52^

Although progesterone has also been linked to mood sensitivtity and emotion-related brain activity, in particular in PMDD,^8^ the absence of a net change in progesterone levels in the current model^19^ prevented us from directly addressing potential contributions from progesterone changes. Yet, within the GnRHa framework we cannot firmly separate effects from different ovarian-steroid hormones, for example, from the early stimulatory phase of the GnRHa intervention. Future studies should address the potential specificity of individual ovarian-steroid hormones in the context.

Our study design does not allow a firm conclusion on whether more complex neurobiological consequences of GnRHa intervention may mediate the effects on insula and amygdala engagement in processing of emotional salient stimuli. Potential mechanistic factors can involve compromised serotonin signalling^17, 53^ or degradation of monoamines in general.^12, 54, 55^ We have previously reported on an interaction between the magnitude of the estradiol decline and increased serotonin transporter availability on the depressive response to GnRHa based on molecular brain imaging data within the present cohort.^19^ It is possible that inter-individual variation in the influence of GnRHa on serotonergic transmission, including effects of the initial stimulatory phase, could explain the proposed variation in estradiol sensitivity. Such a mechanism may well include serotonergic influence on amygdala function,^56^ and will be further pursued in future studies. Further, one might speculate that GnRHa could have a direct effect on insula and/or amygdala activity. However, this is considered unlikely since GnRH receptors are primarily expressed within the Hypothalamus−Pituitary−Gonadal axis and particularly not in insula.^57^ Finally, it should be kept in mind that this study has not yet been replicated.

In conclusion, our data demonstrate that enhanced recruitment of the anterior insula and amygdala during emotional processing have a role in the mechanism by which sex-hormone fluctuations trigger depressive symptoms, which may correspond to the hypersensitivity to emotional stimuli frequently experienced by women in perimenopause or the immediate postpartum period. These findings may provide a rationale for future testing of preventive strategies in high-risk individuals, for example, by thorough monitoring of mental well-being during perimenopause and in the immediate and early postpartum phase, and, to the extent that extreme high-risk individuals can be identified, possibly shorter term estradiol treatment in phases where women decline rapidly or fluctuate in ovarian sex-steroid levels.

## Figures and Tables

**Table 1:** Relationship: brain activity and GnRHa-induced depressive symptoms

*Contrast*	*Region*	*Laterality*	P^a^-*value*	Z-*score*	x	y	z
*Correlation between increase in activity and increase in depressive symptoms*							
F−N	Insula	L	*P*<0.05	3.6	−32	16	0
A−N	Insula	L	*P*_unc_<0.001	3.1	−26	26	−2
		R	*P*_unc_<0.001	3.3	34	32	4
H−N	Insula	L	*P*_unc_<0.001	3.7	−26	22	−2
		R	*P*<0.001	4.0	34	32	6
H−N	Amygdala	R	*P*_unc_<0.001	3.3	28	4	−16

*Correlation between activity post-GnRHa and increase in depressive symptoms*							
F−N	Insula	L	*P*<0.001	4.1	−34	16	−*2*
		R	*P*<0.05	4.1	32	36	0
A−N	Insula	L	*P*_unc_<0.001	3.3	−26	26	−4
		R	*P*_unc_<0.001	3.6	32	24	0
H−N	Insula	L	*P*<0.001	4.4	−26	22	−2
		R	*P*<0.001	4.0	32	22	−2
F−N	Amygdala	L	*P*<0.05	3.5	−24	2	−18
H−N	Amygdala	L	*P*<0.05	3.7	−26	0	−16
		R	*P*<0.05	3.5	28	2	−14

*Correlation between activity post-GnRHa and depressive symptoms post-GnRHa*							
F−N	Insula	L	*P*<0.001	4.8	−34	18	−*6*
		R	*P*<0.001	4.1	48	12	−4
A−N	Insula	L	*P*<0.05	4.1	−26	24	2
		R	*P*_unc_<0.001	3.3	32	22	−2
H−N	Insula	L	*P*<0.001	4.8	−38	−4	−10
		R	*P*<0.001	4.7	48	6	−6
F−N	Amygdala	L	*P*<0.001	3.8	−24	2	−18
H−N	Amygdala	L	*P*<0.05	3.7	−16	−4	−18
H−N	ACC supragenual		*P*<0.05	3.7	2	24	24

Abreviations: A, angry; ACC, anterior cingulate cortex; F, fearful; GnRHa, gonadotropin-releasing hormone agonist; H, happy; N, neutral; unc, uncorrected.

a *P*-values are family wise error-corrected at cluster-level using small volume correction if not otherwise stated.

## References

[bib1] Wittchen HU, Jacobi F, Rehm J, Gustavsson A, Svensson M, Jönsson B et al. The size and burden of mental disorders and other disorders of the brain in Europe 2010. Eur Neuropsychopharmacol 2011; 21: 655–679. 21896369 10.1016/j.euroneuro.2011.07.018

[bib2] Gutiérrez-Lobos K, Scherer M, Anderer P, Katschnig H. The influence of age on the female/male ratio of treated incidence rates in depression. BMC Psychiatry 2002; 2: 3. 11860609 10.1186/1471-244X-2-3 PMC65549

[bib3] Ryan J, Burger HG, Szoeke C, Lehert P, Ancelin M-L, Henderson VW et al. A prospective study of the association between endogenous hormones and depressive symptoms in postmenopausal women. Menopause 2009; 16: 509–517. 19169164 10.1097/gme.0b013e31818d635f PMC2814239

[bib4] Freeman EW, Sammel MD, Lin H, Nelson DB. Associations of hormones and menopausal status with depressed mood in women with no history of depression. Arch Gen Psychiatry 2006; 63: 375–382. 16585466 10.1001/archpsyc.63.4.375

[bib5] Groenewold NA, Opmeer EM, de Jonge P, Aleman A, Costafreda SG. Emotional valence modulates brain functional abnormalities in depression: evidence from a meta-analysis of fMRI studies. Neurosci Biobehav Rev 2013; 37: 152–163. 23206667 10.1016/j.neubiorev.2012.11.015

[bib6] Gingnell M, Morell A, Bannbers E, Wikström J, Sundström Poromaa I. Menstrual cycle effects on amygdala reactivity to emotional stimulation in premenstrual dysphoric disorder. Horm Behav 2012; 62: 400–406. 22814368 10.1016/j.yhbeh.2012.07.005

[bib7] Moses-Kolko EL, Perlman SB, Wisner KL, James J, Saul AT, Phillips ML. Abnormally reduced dorsomedial prefrontal cortical activity and effective connectivity with amygdala in response to negative emotional faces in postpartum depression. Am J Psychiatry 2010; 167: 1373–1380. 20843875 10.1176/appi.ajp.2010.09081235 PMC3293151

[bib8] Toffoletto S, Lanzenberger R, Gingnell M, Sundström-Poromaa I, Comasco E. Emotional and cognitive functional imaging of estrogen and progesterone effects in the female human brain: a systematic review. Psychoneuroendocrinology 2014; 50: 28–52. 25222701 10.1016/j.psyneuen.2014.07.025

[bib9] Rupp HA, James TW, Ketterson ED, Sengelaub DR, Janssen E, Heiman JR. Neural activation in the orbitofrontal cortex in response to male faces increases during the follicular phase. Horm Behav 2009; 56: 66–72. 19306881 10.1016/j.yhbeh.2009.03.005 PMC2742477

[bib10] Derntl B, Windischberger C, Robinson S, Lamplmayr E, Kryspin-Exner I, Gur RC et al. Facial emotion recognition and amygdala activation are associated with menstrual cycle phase. Psychoneuroendocrinology 2008; 33: 1031–1040. 18675521 10.1016/j.psyneuen.2008.04.014 PMC7437605

[bib11] Andreano JM, Cahill L. Menstrual cycle modulation of medial temporal activity evoked by negative emotion. Neuroimage 2010; 53: 1286–1293. 20637290 10.1016/j.neuroimage.2010.07.011 PMC3376005

[bib12] Barth C, Villringer A, Sacher J. Sex hormones affect neurotransmitters and shape the adult female brain during hormonal transition periods. Front Neurosci 2015; 9: 37. 25750611 10.3389/fnins.2015.00037 PMC4335177

[bib13] Petersen N, Cahill L. Amygdala reactivity to negative stimuli is influenced by oral contraceptive use. Soc Cogn Affect Neurosci 2015; 10: 1266–1272. 25688096 10.1093/scan/nsv010 PMC4560944

[bib14] Munafò MR, Brown SM, Hariri AR. Serotonin transporter (5-HTTLPR) genotype and amygdala activation: a meta-analysis. Biol Psychiatry. 2008; 63: 852–857. 17949693 10.1016/j.biopsych.2007.08.016 PMC2755289

[bib15] Passamonti L, Crockett MJ, Apergis-Schoute AM, Clark L, Rowe JB, Calder AJ et al. Effects of acute tryptophan depletion on prefrontal-amygdala connectivity while viewing facial signals of aggression Biol Psychiatry.; 2012; 71: 36–43. 21920502 10.1016/j.biopsych.2011.07.033 PMC3368260

[bib16] Norbury R, Taylor MJ, Selvaraj S, Murphy SE, Harmer CJ, Cowen PJ. Short-term antidepressant treatment modulates amygdala response to happy faces. Psychopharmacology (Berl) 2009; 206: 197–204. 19585106 10.1007/s00213-009-1597-1

[bib17] Bethea CL, Pecins-Thompson M, Schutzer WE, Gundlah C, Lu ZN. Ovarian steroids and serotonin neural function. Mol Neurobiol 1998; 18: 87–123. 10065876 10.1007/BF02914268

[bib18] Munk-Olsen T, Laursen TM, Pedersen CB, Mors O, Mortensen PB. New parents and mental disorders: a population-based register study. JAMA 2006; 296: 2582–2589. 17148723 10.1001/jama.296.21.2582

[bib19] Frokjaer VG, Pinborg A, Holst KK, Overgaard A, Henningsson S, Heede M et al. Role of serotonin transporter changes in depressive responses to sex-steroid hormone manipulation: a positron emission tomography study. Biol Psychiatry 2015; 78: 534–543. 26004162 10.1016/j.biopsych.2015.04.015

[bib20] Hornboll B, Macoveanu J, Rowe J, Elliott R, Paulson OB, Siebner HR et al. Acute serotonin 2A receptor blocking alters the processing of fearful faces in the orbitofrontal cortex and amygdala. J Psychopharmacol 2013; 27: 903–914. 23824248 10.1177/0269881113494106 PMC4606977

[bib21] Deichmann R, Gottfried J, Hutton C, Turner R. Optimized EPI for fMRI studies of the orbitofrontal cortex. Neuroimage 2003; 19: 430–441. 12814592 10.1016/s1053-8119(03)00073-9

[bib22] Friston KJ, Williams S, Howard R, Frackowiak RS, Turner R. Movement-related effects in fMRI time-series. Magn Reson Med 1996; 35: 346–355. 8699946 10.1002/mrm.1910350312

[bib23] Glover GH, Li TQ, Ress D. Image-based method for retrospective correction of physiological motion effects in fMRI: RETROICOR. Magn Reson Med 2000; 44: 162–167. 10893535 10.1002/1522-2594(200007)44:1<162::aid-mrm23>3.0.co;2-e

[bib24] Plichta MM, Schwarz AJ, Grimm O, Morgen K, Mier D, Haddad L et al. Test-retest reliability of evoked BOLD signals from a cognitive-emotive fMRI test battery. Neuroimage 2012; 60: 1746–1758. 22330316 10.1016/j.neuroimage.2012.01.129

[bib25] Johnstone T, Somerville LH, Alexander AL, Oakes TR, Davidson RJ, Kalin NH et al. Stability of amygdala BOLD response to fearful faces over multiple scan sessions. Neuroimage 2005; 25: 1112–1123. 15850729 10.1016/j.neuroimage.2004.12.016

[bib26] Maldjian JA, Laurienti PJ, Kraft RA, Burdette JH. An automated method for neuroanatomic and cytoarchitectonic atlas-based interrogation of fMRI data sets. Neuroimage 2003; 19: 1233–1239. 12880848 10.1016/s1053-8119(03)00169-1

[bib27] Gingnell M, Engman J, Frick A, Moby L, Wikström J, Fredrikson M et al. Oral contraceptive use changes brain activity and mood in women with previous negative affect on the pill—a double-blinded, placebo-controlled randomized trial of a levonorgestrel-containing combined oral contraceptive. Psychoneuroendocrinology 2013; 38: 1133–1144. 23219471 10.1016/j.psyneuen.2012.11.006

[bib28] Pezawas L, Meyer-Lindenberg A, Drabant EM, Verchinski BA, Munoz KE, Kolachana BS et al. 5-HTTLPR polymorphism impacts human cingulate-amygdala interactions: a genetic susceptibility mechanism for depression. Nat Neurosci 2005; 8: 828–834. 15880108 10.1038/nn1463

[bib29] Fusar-Poli P, Placentino A, Carletti F, Landi P, Allen P, Surguladze S et al. Functional atlas of emotional faces processing: a voxel-based meta-analysis of 105 functional magnetic resonance imaging studies. J Psychiatry Neurosci 2009; 34: 418–432. 19949718 PMC2783433

[bib30] Eippert F, Veit R, Weiskopf N, Erb M, Birbaumer N, Anders S. Regulation of emotional responses elicited by threat-related stimuli. Hum Brain Mapp 2007; 28: 409–423. 17133391 10.1002/hbm.20291 PMC6871321

[bib31] Freeman EW, Sammel MD, Lin H, Nelson DB. Associations of hormones and menopausal status with depressed mood in women with no history of depression. Arch Gen Psychiatry 2013; 63: 375–382. 10.1001/archpsyc.63.4.375 16585466

[bib32] Mehta D, Newport DJ, Frishman G, Kraus L, Rex-Haffner M, Ritchie JC et al. Early predictive biomarkers for postpartum depression point to a role for estrogen receptor signaling. Psychol Med 2014; 44: 2309–2322. 24495551 10.1017/S0033291713003231

[bib33] Guintivano J, Arad M, Gould TD, Payne JL, Kaminsky ZA. Antenatal prediction of postpartum depression with blood DNA methylation biomarkers. Mol Psychiatry 2014; 19: 560–567. 23689534 10.1038/mp.2013.62 PMC7039252

[bib34] Etkin A, Wager TD. Functional neuroimaging of anxiety: a meta-analysis of emotional processing in PTSD, social anxiety disorder, and specific phobia. Am J Psychiatry 2007; 164: 1476–1488. 17898336 10.1176/appi.ajp.2007.07030504 PMC3318959

[bib35] Yacubian J, Gla J, Schroeder K, Sommer T, Braus DF, Bu C. Dissociable systems for gain- and loss-related value predictions and errors of prediction in the human brain. J Neurosci 2006; 26: 9530–9537. 16971537 10.1523/JNEUROSCI.2915-06.2006 PMC6674602

[bib36] Menon V, Uddin LQ. Saliency, switching, attention and control: a network model of insula function. Brain Struct Funct 2010; 214: 655–667. 20512370 10.1007/s00429-010-0262-0 PMC2899886

[bib37] Zhong M, Wang X, Xiao J, Yi J, Zhu X, Liao J et al. Amygdala hyperactivation and prefrontal hypoactivation in subjects with cognitive vulnerability to depression. Biol Psychol 2011; 88: 233–242. 21878364 10.1016/j.biopsycho.2011.08.007

[bib38] Mitterschiffthaler MT, Kumari V, Malhi GS, Brown RG, Giampietro VP, Brammer MJ et al. Neural response to pleasant stimuli in anhedonia: an fMRI study. Neuroreport 2003; 14: 177–182. 12598724 10.1097/00001756-200302100-00003

[bib39] Modinos G, Mechelli A, Pettersson-Yeo W, Allen P, McGuire P, Aleman A. Pattern classification of brain activation during emotional processing in subclinical depression: psychosis proneness as potential confounding factor. PeerJ 2013; 1: e42. 23638379 10.7717/peerj.42 PMC3629065

[bib40] Suslow T, Konrad C, Kugel H, Rumstadt D, Zwitserlood P, Schöning S et al. Automatic mood-congruent amygdala responses to masked facial expressions in major depression. Biol Psychiatry.; 2010; 67: 155–160. 19748075 10.1016/j.biopsych.2009.07.023

[bib41] Townsend JD, Eberhart NK, Bookheimer SY, Eisenberger NI, Foland-Ross LC, Cook IA et al. fMRI activation in the amygdala and the orbitofrontal cortex in unmedicated subjects with major depressive disorder. Psychiatry Res 2010; 183: 209–217. 20708906 10.1016/j.pscychresns.2010.06.001 PMC3382985

[bib42] Protopopescu X, Pan H, Altemus M, Tuescher O, Polanecsky M, Mcewen B et al. Orbitofrontal cortex activity related to emotional processing changes across the menstrual cycle. Proc Natl Acad Sci USA 2005; 102: 16060–16065. 16247013 10.1073/pnas.0502818102 PMC1276043

[bib43] Bannbers E, Gingnell M, Engman J, Morell A, Sylvén S, Skalkidou A et al. Prefrontal activity during response inhibition decreases over time in the postpartum period. Behav Brain Res 2013; 241: 132–138. 23238040 10.1016/j.bbr.2012.12.003

[bib44] O'Hara MW, Wisner KL. Perinatal mental illness: definition, description and aetiology. Best Pract Res Clin Obstet Gynaecol 2014; 28: 3–12. 24140480 10.1016/j.bpobgyn.2013.09.002 PMC7077785

[bib45] Sander D, Grafman J, Zalla T. The human amygdala: an evolved system for relevance detection. Rev Neurosci 2003; 14: 303–316. 14640318 10.1515/revneuro.2003.14.4.303

[bib46] Ousdal OT, Reckless GE, Server A, Andreassen OA, Jensen J. Effect of relevance on amygdala activation and association with the ventral striatum. Neuroimage 2012; 62: 95–101. 22546319 10.1016/j.neuroimage.2012.04.035

[bib47] Wright P, Liu Y. Neutral faces activate the amygdala during identity matching. Neuroimage 2006; 29: 628–636. 16143545 10.1016/j.neuroimage.2005.07.047

[bib48] Winston JS, O'Doherty J, Kilner JM, Perrett DI, Dolan RJ. Brain systems for assessing facial attractiveness. Neuropsychologia 2007; 45: 195–206. 16828125 10.1016/j.neuropsychologia.2006.05.009

[bib49] Gottfried JA, O'Doherty J, Dolan RJ. Encoding predictive reward value in human amygdala and orbitofrontal cortex. Science 2003; 301: 1104–1107. 12934011 10.1126/science.1087919

[bib50] Ossewaarde L, Hermans EJ, van Wingen GA, Kooijman SC, Johansson I-M, Bäckström T et al. Neural mechanisms underlying changes in stress-sensitivity across the menstrual cycle. Psychoneuroendocrinology 2010; 35: 47–55. 19758762 10.1016/j.psyneuen.2009.08.011

[bib51] Sheline YI, Barch DM, Donnelly JM, Ollinger JM, Snyder AZ, Mintun MA. Increased amygdala response to masked emotional faces in depressed subjects resolves with antidepressant treatment: an fMRI study. Biol Psychiatry 2001; 50: 651–658. 11704071 10.1016/s0006-3223(01)01263-x

[bib52] Victor Ta, Furey ML, Fromm SJ, Ohman A, Drevets WC. Relationship between amygdala responses to masked faces and mood state and treatment in major depressive disorder. Arch Gen Psychiatry 2010; 67: 1128–1138. 21041614 10.1001/archgenpsychiatry.2010.144 PMC3253452

[bib53] Gundlah C, Pecins-Thompson M, Schutzer WE, Bethea CL. Ovarian steroid effects on serotonin 1A, 2A and 2C receptor mRNA in macaque hypothalamus. Brain Res Mol Brain Res 1999; 63: 325–339. 9878811 10.1016/s0169-328x(98)00295-2

[bib54] Sacher J, Rekkas PV, Wilson AA, Houle S, Romano L, Hamidi J et al. Relationship of monoamine oxidase-A distribution volume to postpartum depression and postpartum crying. Neuropsychopharmacology 2015; 40: 429–435. 25074638 10.1038/npp.2014.190 PMC4443957

[bib55] Rekkas PV, Wilson AA, Lee VWH, Yogalingam P, Sacher J, Rusjan P et al. Greater monoamine oxidase a binding in perimenopausal age as measured with carbon 11-labeled harmine positron emission tomography. JAMA Psychiatry 2014; 71: 873–879. 24898155 10.1001/jamapsychiatry.2014.250 PMC4942269

[bib56] Fisher PM, Haahr ME, Jensen CG, Frokjaer VG, Siebner HR, Knudsen GM. Fluctuations in [^11^C]SB207145 PET binding associated with change in threat-related amygdala reactivity in humans. Neuropsychopharmacology 2015; 40: 1510–1518. 25560201 10.1038/npp.2014.339 PMC4397409

[bib57] Skinner DC, Albertson AJ, Navratil A, Smith A, Mignot M, Talbott H et al. Effects of gonadotrophin-releasing hormone outside the hypothalamic-pituitary-reproductive axis. J Neuroendocrinol 2009; 21: 282–292. 19187469 10.1111/j.1365-2826.2009.01842.x PMC2669307

